# An Automated Microfluidic Analyzer for *In Situ* Monitoring of Total Alkalinity

**DOI:** 10.1021/acssensors.2c02343

**Published:** 2023-01-05

**Authors:** Colin Sonnichsen, Dariia Atamanchuk, Andre Hendricks, Sean Morgan, James Smith, Iain Grundke, Edward Luy, Vincent Joseph Sieben

**Affiliations:** † Dartmouth Ocean Technologies Inc., 25 Parker Street, Suite 202, Dartmouth, Nova ScotiaB2Y 4T5, Canada; ‡ Dept. of Electrical and Computer Engineering, Dalhousie University, 1360 Barrington Street, Halifax, Nova ScotiaB3H 4R2, Canada; § Dept. of Oceanography, Dalhousie University, 1355 Oxford Street, Halifax, Nova ScotiaB3H 4R2, Canada

**Keywords:** microfluidics, ocean sensors, alkalinity, automated, carbon, environmental monitoring

## Abstract

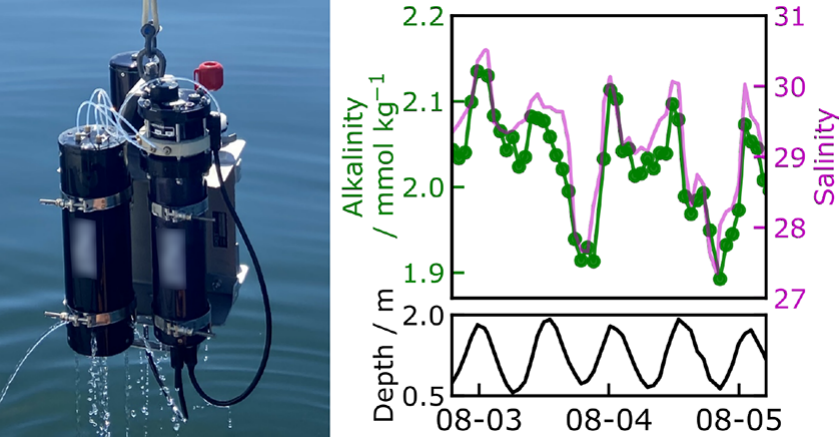

We have designed, built, tested, and deployed an autonomous *in situ* analyzer for seawater total alkalinity. Such analyzers are required to understand the ocean carbon cycle, including anthropogenic carbon dioxide (CO_2_) uptake and for mitigation efforts via monitoring, reporting, and verification of carbon dioxide removal through ocean alkalinity enhancement. The microfluidic nature of our instrument makes it relatively lightweight, reagent efficient, and amenable for use on platforms that would carry it on long-term deployments. Our analyzer performs a series of onboard closed-cell titrations with three independent stepper-motor driven syringe pumps, providing highly accurate mixing ratios that can be systematically swept through a range of pH values. Temperature effects are characterized over the range 5–25 °C allowing for field use in most ocean environments. Each titration point requires approximately 170 μL of titrant, 830 μL of sample, 460 J of energy, and a total of 105 s for pumping and optical measurement. The analyzer performance is demonstrated through field data acquired at two sites, representing a cumulative 25 days of operation, and is evaluated against laboratory measurements of discrete water samples. Once calibrated against onboard certified reference material, the analyzer showed an accuracy of −0.17 ± 24 μmol kg^–1^. We further report a precision of 16 μmol kg^–1^, evaluated on repeated *in situ* measurements of the aforementioned certified reference material. The total alkalinity analyzer presented here will allow measurements to take place in remote areas over extended periods of time, facilitating affordable observations of a key parameter of the ocean carbon system with high spatial and temporal resolution.

Geological records show that, historically, the global ocean has played a central role in regulating Earth’s climate via air–sea exchange of carbon dioxide (CO_2_) at the ocean surface.^1^ Once absorbed by the ocean, the resulting carbonic acid (CO_2_*) would be neutralized by alkaline substances delivered to the ocean by rivers.^2^ Therefore, available geological data demonstrate how ocean alkalinity exerts major control on the ocean’s ability to mitigate the effects of increased atmospheric CO_2_.

To keep the global temperature rise to less than 2 °C by the year 2100, the representative concentration pathway (RCP) 2.6 explicitly requires negative emission technologies to remove CO_2_ from the atmosphere. One such technology, ocean alkalinity enhancement (OAE), allows direct capture from the atmosphere and storage in the deep ocean for many thousands of years.^3,4^ OAE acts to increase seawater pH, shifting the ocean carbonate system away from CO_2_* toward dissolved inorganic carbon in the form of carbonate and bicarbonate ions, allowing more CO_2_ to be taken up from the atmosphere.^5,6^ OAE is thus an accelerated imitation of the natural regulatory pathway employed by the ocean in face of high atmospheric CO_2_. For OAE to be scaled, monetized, and monitored, the additional CO_2_ flux and sequestered carbon needs to be shown and quantified. For this aim, and to understand anthropogenic effects on ocean alkalinity, it will be necessary to increase spatial and temporal frequency of observations of total alkalinity when testing OAE or implementing it at industrial scales for climate change mitigation.

Total alkalinity is a measure of the acid neutralizing capacity of a sample of seawater, typically derived by titration through a series of strong acid additions to a water sample while measuring pH.^7^ A series of acid additions and pH measurements builds a titration curve, which is used to determine the sample alkalinity based on knowledge of the acid–base systems in seawater. The progress of titration can be monitored in a closed^8−12^ or open cell^13−15^ using an indicating dye^16^ or calibrated electrodes.^17,18^ The titrating acid can be injected as a fluid or generated through an anodic current pulse in an electrochemical cell.^19−21^ A handful of benchtop and submersible devices exist, aiming to lower cost and increase coverage of alkalinity measurements. Notable among these instruments are the submersible autonomous moored instrument for alkalinity (SAMI-alk)^10^ and the ion-selective field effect transistors (ISFETs) based on chronopotientometry.^21^ The SAMI-alk enables month-long deployments and can measure alkalinity with a precision of ±4.7 mol kg^–1^ and an accuracy of −2.2 μmol kg^-1 10^. The SAMI-alk is housed in a pressure case approximately 96.5 cm long and 16.5 cm in diameter and is powered by 18 D-cell batteries, sufficient for 2400 samples.^9^ SAMI-alk measurements require ∼12 min, 4.5 mL of titrant, and 80 mL of sample. ISFETs can measure alkalinity and pH simultaneously within one small device at a relatively high frequency;^19,21^ the autonomous sensor of Briggs et al. recorded alkalinity and pH every 2 min in a 100 mL flow cell.^22^ By measuring two components of the seawater carbon system, these electrochemical devices allow for calculation of *p*CO_2_ and dissolved inorganic carbon (DIC) in real time. Although chronopotentiometry shows great potential, ISFETs face encapsulation challenges. Shipboard and lab-based instruments also exist, with strong performance in terms of accuracy and precision,^12,13,15^ for example, the CONTROS FIA system.^13^ The National Oceanographic Center (NOC) in Southampton, UK, has also developed a microfluidic *in situ* alkalinity analyzer with an estimated precision of 5 μmol kg^–1^.^23^ This device, or one similar to it, was used in a controlled CO_2_ release experiment.^24^ Despite the rapid progress in alkalinity analyzer development, encouraged and sustained by the demand for carbon cycle and OAE studies, there remains a need for a rugged, miniaturized, cost-effective autonomous instrument. To the authors’ knowledge, field ruggedness has been a particular challenge in instrument development, with the SAMI-alk system reporting field data for no more than 23 consecutive days^9^ and the ISFET systems showing 6 days of continuous field data.^22^

Microfluidic *in situ* platforms are a proven technology for autonomous sensors in ocean environments.^25^ Microfluidics allows for small reagent volumes and low power consumption, necessary criteria for minimizing payload size on any deployment. Low fluid consumption further allows for the analysis of onboard calibration standards. The features of microfluidic platforms often make it possible to fully replicate and automate the gold-standard wet-chemistry procedures normally carried out by highly trained personnel in shore-based laboratories. Moreover, the reproducible flow conditions in microfluidic devices makes for reproducible experiments. Microfluidics therefore reduces the cost per sample analyzed without compromising on data quality. Microfluidic sensors have been developed to analyze orthophosphate,^26,27^ nitrate and nitrite,^28,29^ iron,^30^ manganese,^31^ pH,^32,33^ and other physical ocean parameters. Microfluidic devices have been deployed to depths of 5000 m,^34^ demonstrating use of the technology in the most demanding ocean environments.

Here, we present a microfluidic total alkalinity analyzer capable of long-term deployments that aims to reduce the cost and increase the coverage of alkalinity sampling. The small physical size and small fluid consumption of this analyzer is afforded by the microfluidic lab-on-chip (LOC) device that handles fluid mixing, chemical reactions, and optical detection. By using independent, stepper motor-driven syringe pumps, we achieve highly repeatable fluid mixing ratios, as opposed to tracer monitored titrations, which rely on absorbance measurements to determine dilution factors.^8^ The platform for our alkalinity analyzer has been through several field trials, tethered to jetties and moored platforms^26^ for up to three months of continuous operation, in estuary and open-ocean type settings. Our optical cell geometry allows for electro-optic elements to be placed independently of the acrylic LOC, no epoxies or optical components are used in LOC fabrication. This independence facilitates LOC fabrication and instrument maintenance; a faulty LOC can be replaced in the field, and LEDs can be swapped to explore different indicating dyes or reactions without replacing the entire acrylic LOC. Using two optical cells, we were further able to compensate the smaller attenuation coefficient of protonated bromocresol green (BCG) with a longer optical path, something that is challenging in single cell geometries. In addition, the analyzer architecture has the flexibility to explore different flow strategies, such as continuous flow, flow injection, or segmented flow analyses.

## Methods

### Reagents

The following reagents were purchased from Fisher Scientific and used without modification: ACS reagent grade sodium chloride (NaCl, >99% purity), analytic grade sodium carbonate (Na_2_CO_3_, >99% purity), bromocresol green sodium salt (BCG), and certified 1.0 N hydrochloric acid (HCl) solution. Unless otherwise noted, all solutions were prepared in artificial seawater (ASW) composed of 0.7 mol kg^–1^ NaCl. ASW backgrounds are necessary to match ionic strengths in the sample and titrant. A stock solution of 100 μmol kg^–1^ BCG was prepared by dissolving 0.0738 g BCG sodium salt in 1.0 L of ASW. This stock solution was used to prepare all titrants. The 0.01 mol kg^–1^ HCl and 20 μmol kg^–1^ BCG titrant used at both field sites was prepared by adding 200 mL of the indicator stock solution and 10 mL of 1.0 N HCl to a 1 L volumetric flask and topping it up with 0.7 mol kg^–1^ ASW.

To prepare internal alkalinity standards, the Na_2_CO_3_ powder was first dried in a vacuum oven at 270 °C for 2.5 h to remove adsorbed water. Na_2_CO_3_ powder was re-dried before every preparation of alkalinity standards. Ten standards with Na_2_CO_3_ concentrations of 769.8 – 1228.6 μmol kg^–1^ in ASW were prepared through direct addition of Na_2_CO_3_ powder to ASW. These standards have alkalinity from 1239.7 to 2457.3 μmol kg^–1^.

For field deployments, freshly prepared acid/indicator titrant and freshly opened certified reference material (CRM), provided by the Prof. Andrew Dickson CRM Laboratory (Scripps Institution of Oceanography, San Diego, USA) were immediately transferred to gas-impermeable bags (Labtainer BioProcess Container).

### Instrument Description

**Figure 1:**
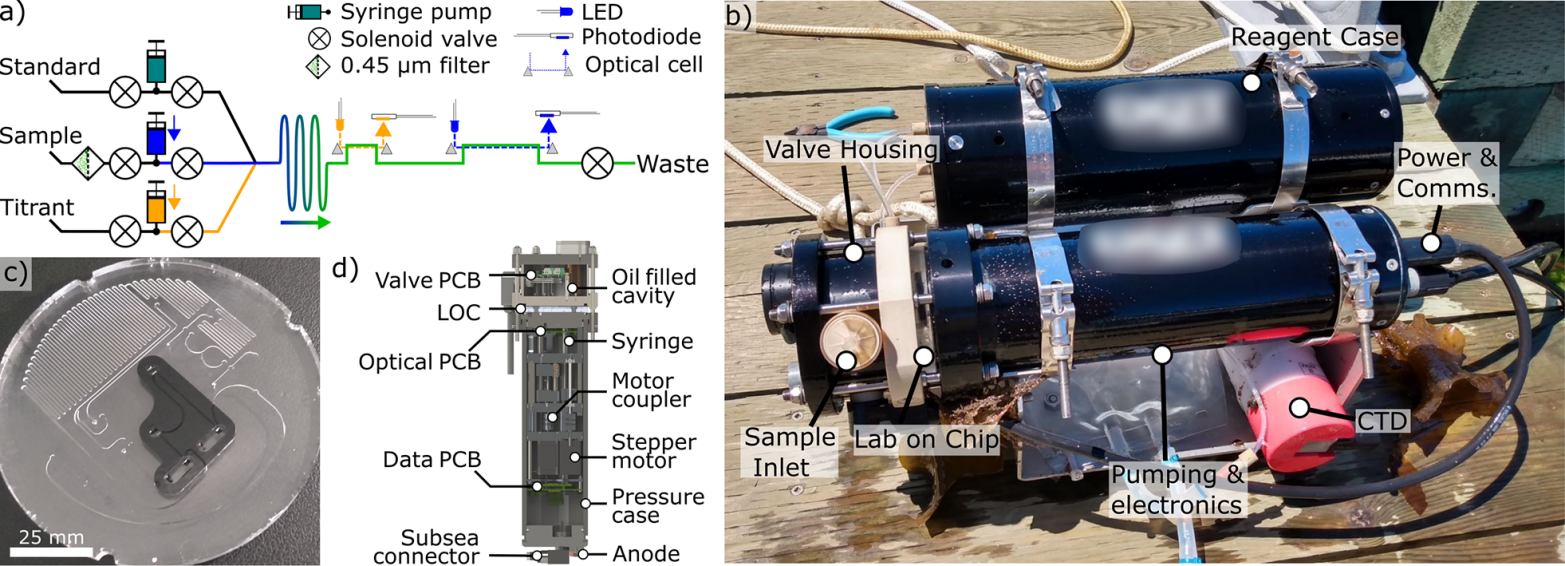
Design of the microfluidic LOC and the total alkalinity analyzer. (a) Fluid schematic showing pumps, valves, optical cells, and optical components. While titrating, each point is built from fresh sample and titrant. Fluid paths during sample pH determination are shown with solid arrows and optical paths with dashed arrows. (b) Photograph of the alkalinity analyzer with a reagent case immediately after COVE jetty experiments, with a co-deployed CTD sensor. (c) Image of the LOC platform in production, showing the channels and inlaid optical cells. This layer is subsequently bonded with a capping layer to form the closed system. (d) Analyzer cross section showing location of major mechanical and electrical components.

Figure 1a shows the fluid schematic of the alkalinity analyzer. Fluid to be measured is pumped either from the environment, after passing through a 0.45 μm filter, or from an onboard standard container. The sample and standard lines use separate syringe pumps to avoid cross-contamination. The sample is mixed with titrant from the acid/indicator pump in a serpentine mixer before entering the optical detection cells. The geometry of the optical cells couples LEDs and photodiodes below the LOC to the measured fluid through total internal reflection at an acrylic–air interface, as described in Luy et al.^35^ Two optical cells placed in series are used to measure the absorbance of the sulfonephthalein indicating dye BCG at two wavelengths, 450 and 620 nm. The optical cells have different path lengths; the protonated form of BCG is measured in a 25.4 mm optical cell at 450 nm, while the deprotonated form is measured at 620 nm in a 10.0 mm optical cell. The ratio of absorbances allows for pH determination through the indicator acid dissociation constant, pK_ind_. The fraction of titrant in solution, and thus the progress of the titration, is determined by the flow ratio between the sample pump and the titrant pump.

The fully assembled and mounted instrument is shown in Figure 1b. The analyzer unit, with the microfluidic LOC, is contained in the lower pressure case in Figure 1b, while the upper free-flooding case contains the acid/indicator titrant and an onboard CRM standard in individual bags. An instrument measuring conductivity, temperature, and depth, CTD (Brevio3 CTD, RBR Ltd., Canada), is co-deployed with our analyzer to log temperature and salinity, which are necessary parameters for the calculation of seawater alkalinity from titration data. For this deployment, power was supplied by an onshore supply. Power and communications go through subsea connector ports on the bottom of the analyzer. The analyzer can also be powered by an external 7.2 V battery pack, housed in a separate pressure case.

The microfluidic LOC fabrication has been described previously.^26,35^ Extruded poly(methyl-methacrylate) (PMMA, commonly known as acrylic) is used throughout the manufacture of these chips. The optical cells are enclosed in a black-tinted PMMA inlay, isolating optical measurements from ambient and scattered light. Channel dimensions are nominally 400×400 μm. The LOCs are produced through a sequence of milling features in the PMMA substrate using a CNC-micromill (LPKF S103), and bonding in a heated press (LPKF MultiPress S). Pieces are cut out of the larger PMMA sheets using an Epilog 50 W laser cutter. To improve the bond strength, PMMA surfaces are exposed to chloroform vapor for 40 s just before going into the heated press.^36^Figure 1c shows an LOC before the final bonding step, with the optical cells near the center of the chip in the black inlay.

Figure 1d is a cross section of the analyzer showing internal components. There are two main parts to this analyzer: an upper assembly containing solenoid valves and a lower assembly containing syringe pumps, optical sensing components, and communication/logging capabilities. The LOC is located between these two main components. Fluidic connections between the LOC and titrant, waste, and standards are made through a PEEK manifold on the bottom of the solenoid valve compartment. After passing through a 0.45 μm filter, the sample flows through a port on the upper assembly, through a solenoid valve, and into the LOC. The overall dimensions of the analyzer are 12 cm in diameter and 40 cm in length.

### Total Alkalinity Determination

Total alkalinity is defined as “the number of moles of hydrogen ion equivalent to the excess of proton acceptors over proton donors in one kilogram of sample” where proton donors are weak acids with pK_a_ > 4.5, and proton acceptors are weak bases with pK_a_ ≤ 4.5.^7^

The development of eqs 1–3 follows closely that of Martz. et al.^8^ A more detailed derivation of eqs 1–3 can be found in the Supporting Information. For seawater samples in normal conditions (negligible concentrations of phosphate, silicate, and other minor acid–base systems including contribution from the organic acids), total alkalinity is defined as follows:
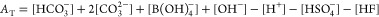
1

If eq 1 is rearranged slightly, and concentrations of individual species are related back to their equilibrium constants, we arrive at eq 2, with the addition of protons donated by an indicating dye:
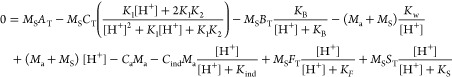
2where *M*_S_ is mass of sample, *M*_a_ is mass of acid, *C*_a_ is titrant acid concentration, *C*_ind_ is titrant indicator concentration, *C*_T_ is the total dissolved inorganic carbon ([CO_2_^*^] + [CO_3_^2–^] + [HCO_3_^–^]), and *B*_T_, *F*_T_, and *S*_T_ are the total borate, fluoride, and sulfate concentrations, respectively. *K_i_* are the various equilibrium constants (*K*_1_ and *K*_2_ are the first and second dissociation constants of the carbonate ion, respectively, *K*_w_ for water, *K*_ind_ for the indicating dye, *K*_S_ for the second dissociation of hydrogen sulfate, *K*_B_ for borate, and *K*_F_ for hydrogen fluoride). All concentrations and equilibrium constants are in units of mol (kg·soln)^–1^.

Dividing eq 2 by the total mass, *M*_S_ + *M*_a_, and substituting in the acid dilution factor *f*_a/i_ = *M*_a_/(*M*_S_ + *M*_a_), alkalinity is related to dilution factors and proton concentrations.
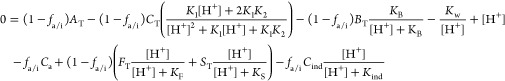
3

A titration curve consists of a set of pH measurements across a range of acid dilution factors. In our microfluidic setup, acid dilution factors are determined by the sample–titrant flow ratio, *f*_a/i_ = *Q*_a_/(*Q*_S_ + *Q*_a_), with *Q*_a_ the volumetric titrant flow rate and *Q*_S_ the volumetric sample flow rate. We are assuming that the sample and titrant have equal density. It should be noted that dilution factors can also be measured optically, by relating optical absorbance on both channels to indicator concentration. For the instrument presented here, the pump-based measure of dilution works best, while other systems have stronger performance using an optical measurement of dilution.^8^ With temperature- and salinity-dependent equilibrium constants defined for seawater taken from the “Guide to Best Practices for Ocean CO_2_ Measurements”,^37^*K*_ind_ taken from Breland and Byrne,^16^ and assuming *F*_T_, *B*_T_, and *S*_T_ are proportional to salinity, the right hand side of eq 3 is minimized in a nonlinear least-squares fashion against *A*_T_ and *C*_T_ to determine total alkalinity. Minimization is performed through Python’s lmfit package; the specific algorithm is Levenberg–Marquardt or damped least-squares. While *C*_T_ is retrieved in this procedure, the uncertainties are generally too large to be of use in an analytical sense.

### Automation Protocol

A titration consists of the following steps: (1) 1 mL of seawater is pumped through the LOC. (2) A blank voltage, *V*_bl_, is recorded over 20 s. (3) Titrant and seawater are flowed simultaneously at a precise ratio, determined by the preset *f*_a/i_, through the LOC. The total volume pumped (sample plus titrant) is 1 mL. The total flow rate, *Q*_S_ + *Q_A_*, is 4 mL min^–1^. (4) A sample voltage, *V*_S_, is measured on both optical paths over 40 s. (5) Steps (3) and (4) are repeated, drawing new sample and titrant at each repetition, to build a titration curve by varying *f*_a/i_ over a preset range.

**Figure 2:**
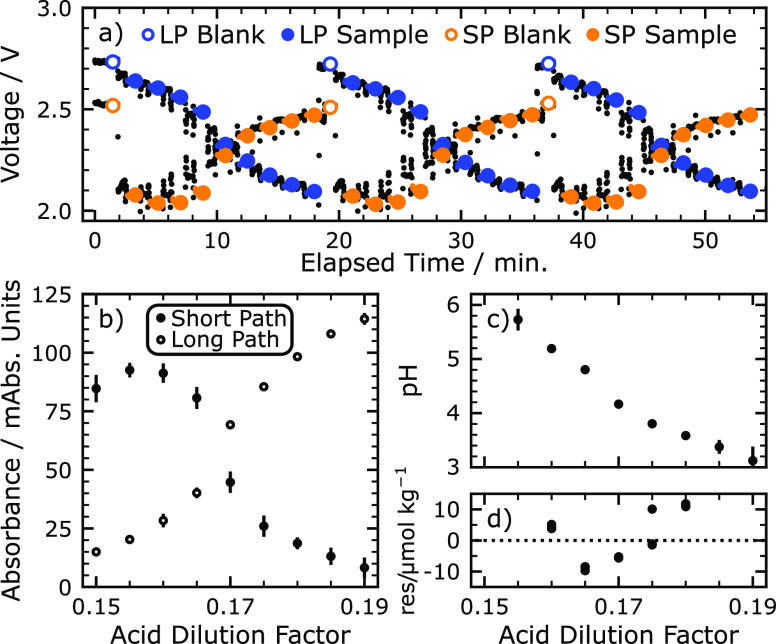
(a) Raw photodiode voltage acquired during three titrations of a Na_2_CO_3_ standard at 15 °C. LP and SP refer to the two optical cells in our device. LP is the long path; SP is the short path. (b) Calculated absorbance on both optical paths from voltages in (a); error bars represent 3σ, or 3 times the standard deviation from the triplicate experiments. The long path records absorbance at 450 nm, and the short path records absorbance at 620 nm. (c) Calculated pH from absorbance data in (b), according to eq 10 in the text. (d) Residuals from the least squares minimization procedure.

The resulting data from this procedure is shown in Figure 2. Photodiodes are recording voltages at a frequency of 1 Hz, and data is tagged depending on analyzer status (pumping, recording blank, recording sample, etc.). All blank, sample, and standard voltages are measured in a static condition, i.e., there are no pump actuations or active fluid movement. The tagged photovoltages through three repetitions of a titration are shown in Figure 2a, for both the long (25.4 mm) and short (10.0 mm) optical paths, at 450 and 620 nm respectively.

At each point of the titration curve, a fresh sample and titrant are introduced to the optical cell. While this protocol is not fluidically or energetically efficient, it greatly simplifies the design and implementation of the alkalinity analyzer. It also means that the analyzer becomes sensitive to short-term variations in alkalinity over the timespan of titration. Each titration point consumes between 140 and 200 μL of titrant and 800 and 860 μL of sample, is measured in 105 s, and consumes 460 J of energy. An alkalinity determination from a set of nine titration points and one blank will consume approximately 8.5 mL of sample or standard, 1.5 mL of titrant, and 4400 J of energy. The entire titration takes about 20 min.

Our protocol is also flexible in the choice of *f*_a/i_ sampled. This flexibility can be exploited to decrease measurement time or change the alkalinity sampling range without changing acid concentrations. For example, if alkalinity is not expected to vary significantly, *f*_a/i_ at extreme ends of the titration curve could be omitted, decreasing sampling time and reducing fluid/energy consumption. This would have no impact on analyzer precision if these extreme ends are consistently outside the pH sensing range of the indicating dye. Alternatively, if alkalinity is especially high or low, the entire range of *f*_a/i_ could be shifted up or down, respectively. Our instrument has the capacity to make these changes autonomously and could programmatically change which *f*_a/i_ to sample even partway through a titration, based on the previous pH measurement. These optimizations may be pursued in a subsequent study.

### Data Analysis

The long optical path monitors absorption from the protonated form of BCG, [HI], at 450 nm, while the short optical path monitors the deprotonated form, [I^–^], at 620 nm. As the titration progresses toward lower pH, absorbance on the long optical path increases while absorbance on the short optical path decreases, as shown in Figure 2b. Absorbance is calculated from the raw data in Figure 2a according to the following equation:
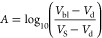
4where *A* is sample absorbance, *V*_bl_ is the blank voltage, and *V*_S_ the sample voltage. *V*_d_ is the dark voltage recorded by the photodiodes when the LEDs are off. The Beer–Lambert law allows absorbance from each optical cell to be related to the concentration of each species.
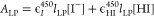
5
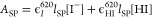
6

In eqs 5 and 6, *A*_SP_ and *A*_LP_ are the absorbance values on the long and short optical paths, respectively, *l*_SP_ and *l*_LP_ are the corresponding path lengths (10.0 and 25.4 mm, respectively), and ϵ_*i*_^λ^ is the molar attenuation coefficients of the two forms of BCG at the wavelength of the probing LED. Equations 5 and 6 allow for the calculation of hydrogen ion concentration in solution through the acid dissociation constant for the reaction [HI] ↔ [H^+^] + [I^–^]. Along with the relation defining pH on the total scale, pH = – log_10_[H^+^], hydrogen ion concentration can be calculated from absorbance measurements of an indicating dye at two wavelengths. Stated in equation form
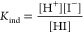
7
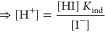
8

Rearranging eqs 5 and 6 to solve for [HI] and [I^–^] and then substituting into eq 8,
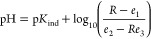
9
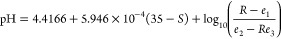
10

In eqs 9 and 10, *R* is the ratio of measured absorbance between the short and long optical paths, *R* = *A*_SP_/*A*_LP_, and the *e_i_* are ratios of attenuation coefficients, taking the different optical path lengths into account. *e*_1_ = ϵ_HI_^620^*l*_SP_/ϵ_HI_^450^*l*_LP_, *e_2_ = ϵ*_*I*_^620^*l*_SP_/ϵ_HI_^450^*l*_LP_, *e*_3_ = ϵ_*I*_^450^*l*_LP_/ϵ_HI_^450^*l*_LP_. The step-wise derivation of eq 9 can be found in the Supporting Information. Due to the use of two different optical paths, the molar absorption coefficients, ϵ_*i*_^λ_*j*_^*l_j_* for each form of BCG had to be measured for our specific optical geometry. These results are summarized in Table 1.

**Table 1:** Molar Absorbance for BCG in the Two-Path Optical Geometry of our Analyzer

LED λ_0_ (nm)	ϵ_HI_*l*(AU/mol)	ϵ*_I_l*(AU/mol)
620	251	38.3 × 10^3^
450	38.7 × 10^3^	6.45 × 10^3^

The molar absorptivities listed in Table 1 lead to *e_i_* ratios that differ from those found in the literature due to the use of two different path lengths in our setup. We use the values *e*_1_ = 0.0065, *e*_2_ = 0.990, and *e*_3_ = 0.167, calculated from the absorptivities in Table 1. Breland and Byrne^16^ reported *e*_1_ = 0.0013, *e*_2_ = 2.3148, and *e*_3_ = 0.1299.Using these values and taking into account our longer path length at 450 nm, we would expect our values to be 0.00052, 0.911, and 0.1299 (the absorbances for the ratio *e*_3_ are along the same optical path). The discrepancy is due in part to our use of LEDs and broad band detection, where Breland and Byrne^16^ made use of a spectrophotometer with wavelength selection.

The absorbance values shown in Figure 2b are used to calculate pH according to eq 10. These pH values versus acid dilution factor are shown in Figure 2c and form a titration curve. As expected, a small standard deviation is observed between the pH values from 4 to 5, the optimal sensing range of the indicating dye. To improve the performance of the least squares minimization, titration points outside the pH range 3.75–5.40 and outside the absorbance range 0.01–0.18 are omitted from the minimization procedure. The pH range was chosen based on the shape and distribution of residuals over many titrations. These residuals can be found in the Supporting Information. The filtered data is used as the input to the minimization of eq 3 with respect to *A*_T_ and *C*_T_. The residuals from this fitting procedure are shown in Figure 2d. If residual values exceed 50 μmol kg^–1^ in the absolute value, the least squares minimization is repeated with those data omitted. For the example shown in Figure 2, the initial set of nine titration points was filtered down to five points as input to the minimization of eq 3. This data analysis determined an alkalinity of 1996 μmol kg^–1^.

### Laboratory Analysis of Discrete Water Samples

To evaluate and compare the analyzer performance to standard lab-based approaches, separate discrete water samples for total alkalinity were collected in duplicates following the standard operating procedures described in Dickson et al.^37^ Water samples were collected in 500 mL borosilicate glass bottles, poisoned with 100 μL of a saturated solution of HgCl_2_, and analyzed in the CERC-Ocean laboratory at Dalhousie University within 4 weeks of collection. Sample alkalinity was measured using an open cell potentiometric titration performed by an automated titration system, which follows the protocol outlined in Dickson et al.^37^ Accuracy of the system was assured through regular analysis of CRM, and the precision measured on CRM duplicates was 1.7 μmol kg^–1^.

## Results

**Figure 3:**
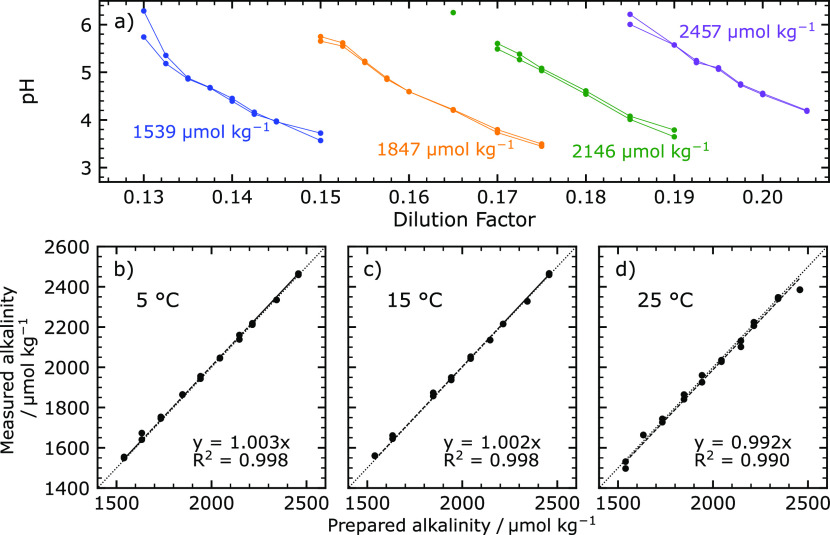
Temperature dependance of alkalinity measurements. (a) Duplicate titration curves from four prepared Na_2_CO_3_ standards, with prepared alkalinity shown by the colored text. These data were obtained at 10 °C. (b–d) Resulting calibration curves at 5, 15, and 25 °C, respectively. Dashed lines are linear fits to the data, and dotted lines are the 1:1 expected relation.

Prior to being deployed *in situ*, temperature effects were characterized. Important uncertainties that arise from variations in temperature are mostly related to the absorbance and equilibrium properties of BCG, i.e., changes in the ϵ_*i*_^λ^ and *K*_ind_ with temperature. Changes in density of the sample and titrant are assumed equal due to their approximately equal ionic strength and equal temperature. Previous works^8,15,16^ have examined indicator changes and compensated for them collectively through a corrected absorbance ratio described by Breland and Byrne:^16^
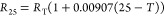
11where *R*_T_ is the measured absorbance ratio, *T* is temperature in °C, and *R*_25_ is the corrected absorbance ratio. While eq 11 has proved to be sufficient for previous analyzers, the temperature range explored by Breland and Byrne^16^ only covered 18–32 °C, suitable for lab- and ship-based instruments but not for *in situ* analyzers. The approach taken in this work was to assume the correction of Breland and Byrne^16^ holds across the entire temperature range of field measurements. This was experimentally verified in the lab, with results shown in Figure 3. Nine standards covering the range 1540–2460 μmol kg^–1^ were prepared using dehydrated Na_2_CO_3_ in 0.7 mol kg^–1^ NaCl ASW. A CRM was also analyzed, CRM batch 202. These 10 solutions were analyzed by our alkalinity analyzer fully submerged in a temperature-controlled bath at 5, 10, 15, 20, and 25 °C. The results of these tests are shown in Figure 3. Additional results can be found in the Supporting Information.

Figure 3a shows a duplicate titration for four of the prepared standards. All titrations in Figure 3a were performed at a temperature of 10 °C. The retrieved alkalinity from all titrations is shown in Figure 3b–d at temperatures of 5, 15, and 25 °C, respectively. Also shown in Figure 3b–d are linear fits to the data as dashed lines, to be compared to the dotted unity line along *y* = *x*. All slopes are between 0.99 and 1.00 (less than 1% difference), indicating a high degree of accuracy within the 5–25 °C temperature range. It is apparent from the data shown in Figure 3 that temperature effects play a minor role at best and are adequately compensated with the correction of eq 11.

**Figure 4:**
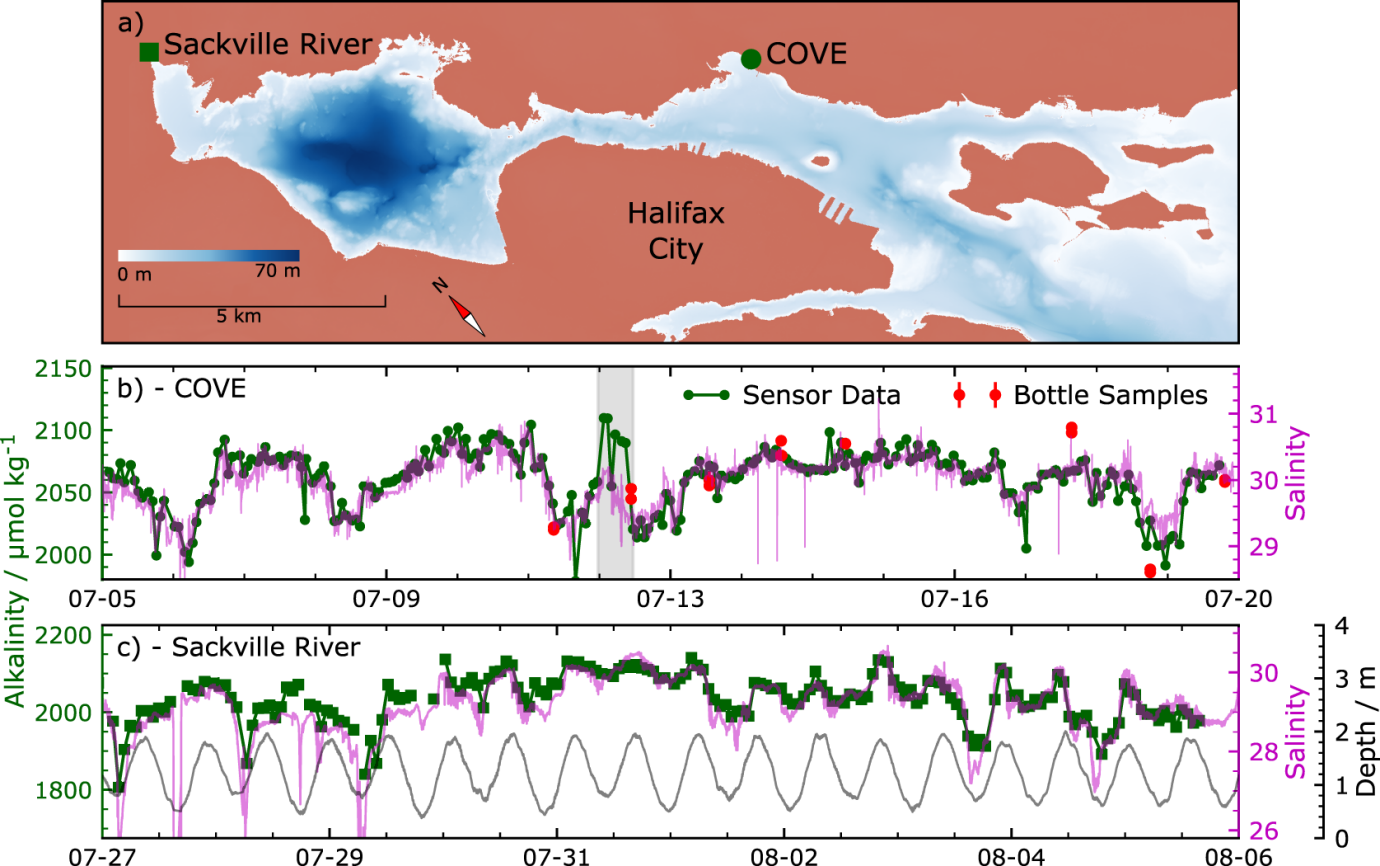
Field data obtained between July 5 and August 6, 2022. (a) Bathymetric map of Halifax Harbor and Bedford Basin on the east coast of Canada. Deployment sites at the COVE jetty (44.66°N, 63.56°W) and at the mouth of the Sackville River (44.73°N, 63.66°W) are marked with a green circle and a green square, respectively. (b) Retrieved alkalinity and salinity data from the COVE jetty site. Bottle samples are marked with red circles. The grayed-out region indicate inferior quality data due to bubble formation in the optical cell. (c) Retrieved alkalinity, salinity, and depth from the Sackville River site.

To evaluate the *in situ* performance of our total alkalinity analyzer, the instrument was deployed at two sites in Halifax Harbor. Figure 4 shows the map for these deployment locations and the data produced by the alkalinity analyzer. For the first deployment, the analyzer was tied off at a jetty at the Center for Ocean Ventures and Entrepreneurship (COVE) in Halifax Harbor, on Canada’s Atlantic coast (44.66°N, 63.56°W, green circle in Figure 4a). The analyzer was deployed with 1 L of acid/indicator titrant and 500 mL of CRM batch 181 as an onboard standard. Power was provided through an onshore power supply. The analyzer maintained a constant depth of ∼1.9 m, while distance from the seabed changed with the tide. The analyzer operated continuously, taking alkalinity measurements approximately every hour. These data are shown in Figure 4b, along with lab-analyzed bottle samples and salinity from a co-deployed CTD. Except for a brief period from 3 AM to 2 PM on June 12, 2022, when an air bubble entered the optical path, the measured alkalinity tracked salinity very closely. Discrete bottle samples were taken in duplicate with a 5 L Niskin bottle on a regular basis; these are shown as red circles in Figure 4b.

To assess the performance of our analyzer in a more dynamic environment with variable total alkalinity, it was deployed at the mouth of the Sackville River (44.73°N, 63.66°W). The Sackville River is the largest freshwater source for Halifax Harbor, and drives much of the circulation within the Bedford Basin.^38^ The site is depicted by the green square in Figure 4a. Sufficient titrant and standard from the first deployment remained for the second deployment, so these reagents were used without modification or replenishment. Power for the analyzer at the Sackville River was provided by an onboard battery pack, providing 78 Ah of charge at 7.2 V from 12 LiSOCl_2_ D-cell batteries. This power supply is sufficient for approximately 450 samples. Data from the Sackville River site is shown in Figure 4c, along with depth and salinity from a co-deployed CTD. The practical salinity showed important variation with tides, some days changing by upward of 2.5 over 6 h. Over the course of the 9 day deployment, alkalinity again tracked salinity closely through large tidal variations.

## Discussion

**Figure 5:**
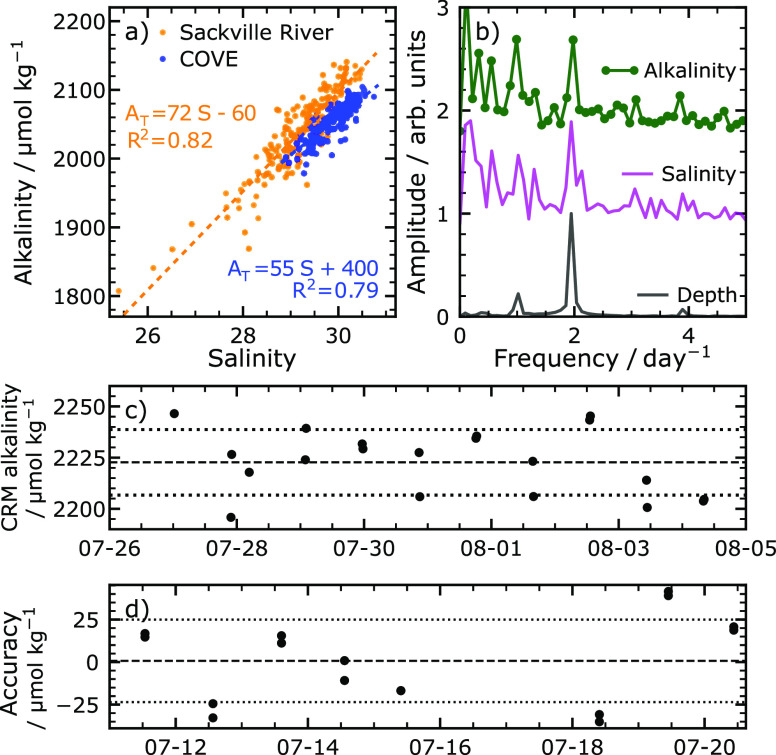
Analysis of deployment data. (a) At both sites, alkalinity is highly correlated with seawater salinity. (b) Fourier transform amplitudes of data obtained at the Sackville River. Tidal variations show as peaks at ∼1.95 and ∼1 day^–1^. (c) Analysis of an onboard certified reference material over the course of the Sackville River deployment. The certified value is shown as a dashed line (batch 181, *A*_T_=2222.7 μmol kg^–1^), and standard error (16 μmol kg^–1^) is shown as dotted lines. (d) Analyzer accuracy when compared to discrete bottle samples obtained at COVE. The dashed line is the average bias (−0.17 μmol kg^–1^), and dotted lines are 1 standard deviation (24 μmol kg^–1^).

The data presented in Figure 4b,c suggest a correlation between salinity and alkalinity at both sites. This correlation is shown and quantified in Figure 5a. Similar correlations have been shown between salinity and alkalinity in freshwater systems^9^ and empirically observed in the open ocean as well.^39^ While correlation coefficients are significant, what is more striking is the difference between the salinity–alkalinity relations between sites. At COVE, far from major freshwater sources, the relationship is described by the linear relation *A*_T_ = 55*S* + 400. At the Sackville River, where alkalinity variations are tidally driven, the relation is modeled by *A*_T_ = 72*S* – 60. In both preceding equations, *S* is salinity on the practical salinity scale, and *A*_T_ is measured in μmol kg^–1^. Particularly interesting in the salinity–alkalinity relationships described above is the *y*-intercept. For the Sackville River, a near-zero *y*-intercept suggests that the Sackville River brings little alkalinity to the Harbor, and alkalinity measured at this site is due to mixing with water from the Bedford Basin. At COVE, located in the inner harbor, alkalinity is more representative of the open ocean.

To examine the strength of tidal effects at the Sackville River site, the depth, salinity, and alkalinity data were Fourier transformed. These data are shown in Figure 5b. There are three peaks in the depth spectrum, with the most important at 1.95 day^–1^ representing the ∼12 h tidal period. Salinity data has a much noisier spectrum, but the peak at 1.95 day^–1^ is quite prominent. Other peaks that appear in the depth spectrum near 1.0 and 3.9 day^–1^ cannot be distinguished from noise in the salinity spectrum. The alkalinity data shows two clear peaks of approximately equal amplitude, one near 1.0 day^–1^ and the second near 2.0 day^–1^. These data would suggest that alkalinity changes are tidally driven this close to the river mouth.

Figure 5c shows the time series of onboard standard measurements from the Sackville River. Standard measurements were taken in duplicate approximately every 21 h. The average standard measurement over the entire deployment was used to correct the alkalinity data using a constant calibration factor. The average standard (CRM) measure was 2209.3 μmol kg^–1^, compared to the certified value of 2222.71 μmol kg^–1^, resulting in a scaling factor of 1.0061 and accuracy of −13±16 μmol kg^–1^ in comparison to onboard standards. All the alkalinity data in Figures 4 and 5 have been multiplied by this scaling factor, including the values shown in Figure 5c. The standard error of the data shown in Figure 5c represents our measure of analyzer precision. This value is 16 μmol kg^–1^. Notably, no correlation between environmental temperature and retrieved standard alkalinity is found (*R*^2^ = 0.15, slope = −2.0 ± 1.1 μmol kg^–1^ °C^–1^, *N* = 20). The lack of correlation supports our use of eq 11 as a temperature correction over a broader temperature range. Analyzer field accuracy is reported as the average difference between standard-corrected analyzer values and the titration result from discrete bottle samples, as in Figure 5d. This value is −0.17 ± 24 μmol kg^–1^. One reason for the somewhat low precision in bottle samples could be the variable alkalinity at our deployment site, both in time and space. Since the analyzer only takes a measurement every hour, there could be up to 30 min between the analyzer reading and the bottle sampling. Analyzer and bottle comparison was made through linear interpolation of the analyzer alkalinity, but this is only valid if alkalinity values are not changing too quickly. Furthermore, the titration process takes in sample at various times over the course of 20 min, which introduces measurement error in dynamic environments. There is also the possibility of vertical gradients in alkalinity over the height of our Niskin sampler.

## Conclusions

The novel total alkalinity analyzer presented here shows satisfactory accuracy and precision over the range 5–25 °C, as demonstrated by consistent *in situ* standard measures, benchtop experiments, and discrete sample comparison. The 1 L acid/indicator bag and 500 mL standard bag were sufficient to run all measurements at two field sites, with two standards measured daily, for a total of 532 sample measurements and 62 standard measurements. This is only possible due to the low reagent consumption of our microfluidic platform. Further field work will focus on using the analyzer in a variety of applications, for example, on tow bodies or seabed platforms. This will be combined with laboratory investigations seeking further improvements to analyzer performance in terms of accuracy, precision, and fluid consumption. We believe our analyzer will enable large-scale carbon monitoring through space and time for researchers while simultaneously contributing to monitoring and verification of carbon dioxide removal through ocean alkalinity enhancement.

## Supporting Information Available

More detailed derivation of eqs 3 and 9, discussion around the choice of pH range, and supplementary data to Figure 3 showing all temperature trials (PDF)
