# Supplementary material for: An Automated Microfluidic Analyzer for In Situ Monitoring of Total Alkalinity
Source: ACS Sens. 2023 Jan 5;8(1):344–52. doi: 10.1021/acssensors.2c02343 (PMC9888396; doi:10.1021/acssensors.2c02343)
Supplement: Supplementary file 1 — se2c02343_si_001.pdf [file se2c02343_si_001.pdf]

Supporting Material to

"An Automated Microfluidic Analyzer for in-situ Monitoring of Total Alkalinity"

Colin Sonnichsen[1,2*], Dariia Atamanchuk[3], Andre Hendricks[2], Sean Morgan[2], James Smith[1], Iain Grundke[1], Edward Luy[1,2], Vincent Joseph Sieben[1,2*]

[1]Dartmouth Ocean Technologies Inc., 25 Parker Street, Suite 202, Dartmouth, Nova Scotia, Canada B2Y 4T5

[2]Dept. of Electrical and Computer Engineering, Dalhousie University, 1360 Barrington Street, Halifax, Nova Scotia, Canada B3H 4R2

[3]Dept. of Oceanography, Dalhousie University, 1355 Oxford Street, Halifax, Nova Scotia, Canada, B3H 4R2

*corresponding author(s): colin.sonnichsen@dal.ca | sieben@dal.ca

Derivation of equations (1) – (3)

Equation (1) relates the principal acid-base systems of seawater to total alkalinity, equation (2) follows from the definition of each species' equilibrium constant, and conservation of species.

Using the relations for sulphate (S1) and (S2), we can solve for terms such as (S3), which are used in equations (2) and (3) in the main text.

$$K_S = \frac{[\mathrm{H}^+][\mathrm{SO}_4^{2-}]}{[\mathrm{HSO}_4^-]} \qquad \text{(S1)}$$

$$S_T = \left[\mathrm{HSO}_4^-\right] + \left[\mathrm{SO}_4^{2-}\right] \qquad \text{(S2)}$$

$$\Rightarrow \left[\mathrm{HSO}_4^-\right] = S_T \frac{[H^+]}{[\mathrm{H}^+] + K_S} \qquad \text{(S3)}$$

A similar development produces the rest of the terms in equation 2. For a mixture of titrant and sample, those terms that are present in the sample (e.g. bicarbonate ions) are multiplied by the sample mass M_S. Those terms that are present in the titrant (e.g. the indicating dye) are multiplied by the titrant mass, M_a. Those terms that are present in both (e.g. hydroxide ion) are multiplied by the total mass, $M_S + M_a$.

This leads to equation (2). Equation (3) is a straightforward division by the total mass and substitution using $f_{ai} = \frac{M_a}{M_S + M_a}$ as described in the main text.

Derivation of equation 9

Equations (5) and (6) in the main text can be rearranged in terms of $[\mathrm{I}^-]$ and $[\mathrm{HI}]$.

$$\left[\mathrm{I}^-\right] = \frac{\epsilon_{HI}^{450} l_{LP} A_{SP} - \epsilon_{HI}^{620} l_{SP} A_{LP}}{\epsilon_I^{620} l_{SP}\, \epsilon_{HI}^{450} l_{LP} - \epsilon_{HI}^{620} l_{SP} \epsilon_I^{450} l_{LP}} \qquad \text{(S4)}$$

$$[\mathrm{HI}] = \frac{\epsilon_I^{620} l_{SP} A_{LP} - \epsilon_I^{450} l_{LP} A_{SP}}{\epsilon_I^{620} l_{SP}\, \epsilon_{HI}^{450} l_{LP} - \epsilon_{HI}^{620} l_{SP} \epsilon_I^{450} l_{LP}} \tag{S5}$$

Taking the ratio of equations (S4) and (S5):

$$\frac{[\mathrm{I}^-]}{[\mathrm{HI}]} = \frac{\epsilon_{HI}^{450} l_{LP} A_{SP} - \epsilon_{HI}^{620} l_{SP} A_{LP}}{\epsilon_I^{620} l_{SP} A_{LP} - \epsilon_I^{450} l_{LP} A_{SP}} \tag{S6}$$

Dividing equation (S6) by $\epsilon_{HI}^{450} l_{LP} A_{LP}$ in the numerator and denominator:

$$\frac{[\mathrm{I}^-]}{[\mathrm{HI}]} = \frac{R - e_1}{e_2 - Re_3} \tag{S7}$$

Where the terms in (S7), as described in the main text, are as follows:

$R = A_{SP}/A_{LP},\; e_1 = \epsilon_{HI}^{620} l_{SP}/\epsilon_{HI}^{450} l_{LP},\;\; e_2 = \epsilon_I^{620} l_{SP}/\epsilon_{HI}^{450} l_{LP},\;\; e_3 = \epsilon_I^{450} l_{LP}/\epsilon_{HI}^{450} l_{LP}$

One arrives at equation (9) in the main text by taking the negative base ten log of equation (8), and using the above relation (S7) with the fact that $pH = -\log_{10}[\mathrm{H}^+]$ and $pK_{ind} = -\log_{10} K_{ind}$.

$$-\log_{10}[\mathrm{H}^+] = -\log_{10}\left(\frac{[\mathrm{HI}]\, K_{ind}}{[\mathrm{I}^-]}\right) \tag{S8}$$

$$\Rightarrow pH = pK_{ind} - \log_{10}\left(\frac{[\mathrm{HI}]}{[\mathrm{I}^-]}\right) \tag{S9}$$

$$= pK_{ind} + \log_{10}\left(\frac{[\mathrm{I}^-]}{[\mathrm{HI}]}\right) \tag{S10}$$

$$= pK_{ind} + \log_{10}\left(\frac{R - e_1}{e_2 - Re_3}\right) \tag{S11}$$

Choice of pH range

The choice of pH range is determined initially by the sensing range of BCG, however it is possible to measure pH over a broader range, certainly 3.5- 6.0. To choose the specific range of pH values to use in minimizing equation (3), we examined the distribution of fit residuals as a function of titration pH. By examining the entire data set from the Sackville River deployment (standards and samples), we decided to use the range of 3.75 - 5.4. This reduced the number of titration points by approximately 40%, but reduced the number of alkalinity results by only 1%. Figure S1 shows data supporting this choice. The entire data set is shown in fig. S1a) and b). In figure S1a) the shape of residuals is not flat, indicating additional pH-dependent processes not captured in equation (3) in the main text. Additionally, in figure S1b), the distribution of residuals is not normal, and has negative skew.

Limiting the minimization of pH value to the range 3.75 - 5.40 had the effect of narrowing the distribution of residuals, flattening the dependence on pH, and removing the negative skew to the

distribution while not significantly reducing the number of measurements. This is shown in figure S1 c) and d)

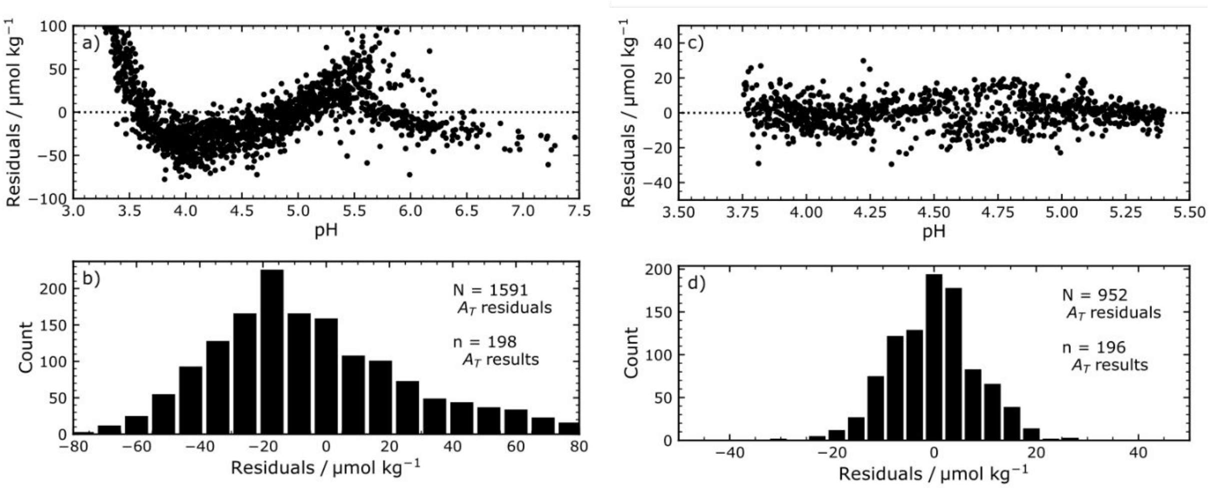

Figure S1: Choice of pH range in determining alkalinity. a) Residuals to minimization of equation (3) as a function of pH, with no constraints on input data. b) Histogram of residuals shown in a). c) Residuals to minimization of equation (3) as a function of pH, where input data is constrained to the pH range 3.75-5.4. d) Histogram of the residuals shown in c).

Supplementary data to figure 3:

As mentioned in the main text, 9 alkalinity standards and one certified reference material were measured at temperatures of 5, 10, 15, 20 and 25°C. All of these data are shown below, in figure S2.

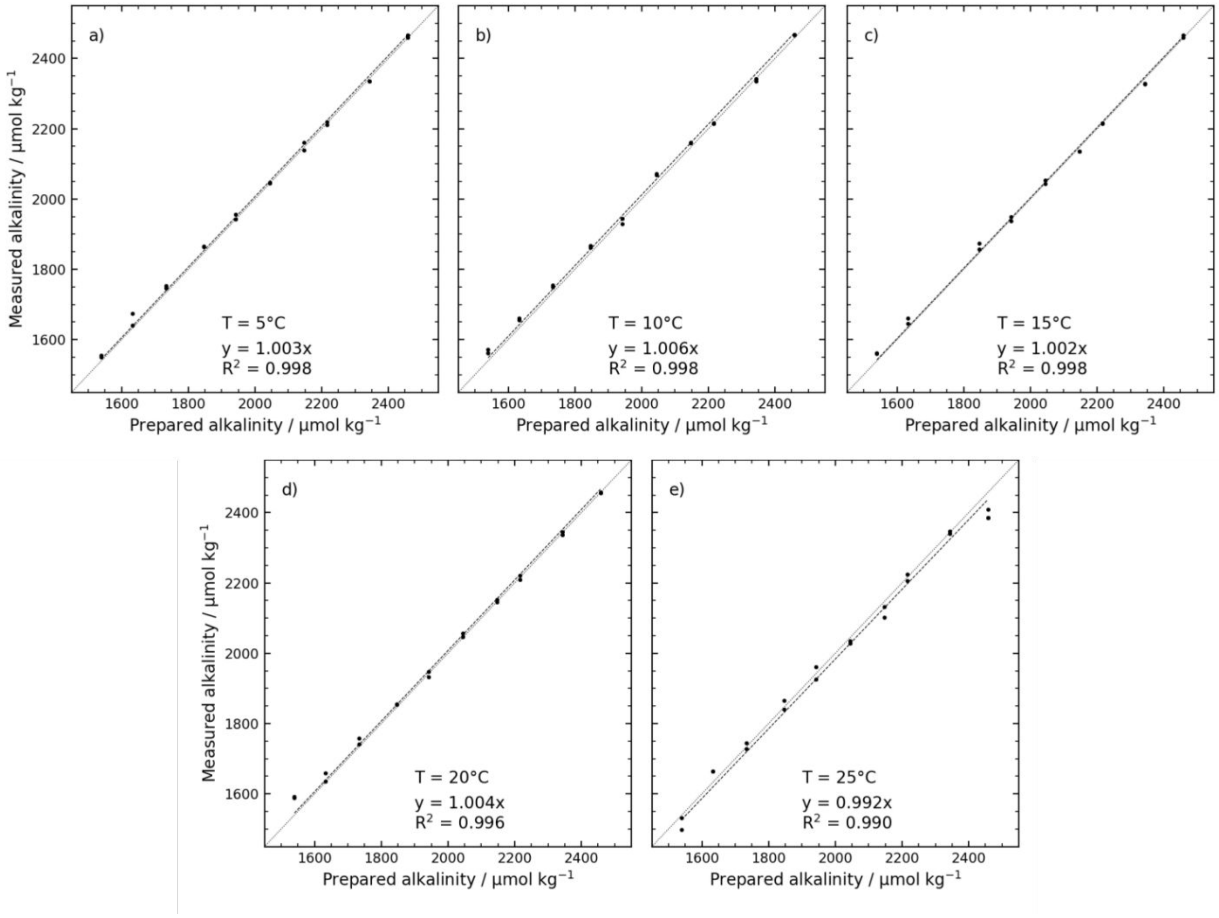

Figure S2: Entire set of alkalinity measurements as a function of temperature, for temperatures a) 5°C, b) 10°C, c) 15°C, d) 20°C, and e) 25°C
